# Human Herpes Virus 8 in HIV-1 infected individuals receiving cancer chemotherapy and stem cell transplantation

**DOI:** 10.1371/journal.pone.0197298

**Published:** 2018-05-10

**Authors:** Louise E. Hogan, Emily Hanhauser, Kristen S. Hobbs, Christine D. Palmer, Yvonne Robles, Stephanie Jost, Anne S. LaCasce, Jeremy Abramson, Ayad Hamdan, Francisco M. Marty, Daniel R. Kuritzkes, Timothy J. Henrich

**Affiliations:** 1 Department of Experimental Medicine, UCSF, San Francisco, CA, United States of America; 2 Division of Infectious Diseases, Brigham and Women's Hospital, Boston, MA, United States of America; 3 Department of Medicine, Harvard Medical School, Boston, MA, United States of America; 4 Ragon Institute of MGH, MIT and Harvard, Cambridge, MA, United States of America; 5 Center for Virology and Vaccine Research, Beth-Israel Deaconess Medical Center, Boston, MA, United States of America; 6 Department of Medicine, Dana-Faber Cancer Institute, Boston, MA, United States of America; 7 Massachusetts General Hospital, Boston, MA, United States of America; Fudan University, CHINA

## Abstract

**Background:**

Human Herpes Virus 8 (HHV8) can cause Kaposi’s Sarcoma (KS) in immunosuppressed individuals. However, little is known about the association between chemotherapy or hematopoietic stem cell transplantation (HSCT), circulating HHV8 DNA levels, and clinical KS in HIV-1-infected individuals with various malignancies. Therefore, we examined the associations between various malignancies, systemic cancer chemotherapy, T cell phenotypes, and circulating HHV8 DNA in 29 HIV-1-infected participants with concomitant KS or other cancer diagnoses.

**Methods:**

We quantified HHV8 plasma viral loads and cell-associated HHV8 DNA and determined the relationship between circulating HHV8 DNA and lymphocyte counts, and markers of early and late lymphocyte activation, proliferation and exhaustion.

**Results:**

There were no significant differences in plasma HHV8 DNA levels between baseline and post-chemotherapy time points or with the presence or absence of clinical KS. However, in two participants circulating HHV8 DNA increased following treatment for KS or HSCT for lymphoma,. We observed an approximately 2-log_10_ reduction in plasma HHV8 DNA in an individual with KS and multicentric Castleman disease following rituximab monotherapy. Although individuals with clinical KS had lower mean CD4^+^ T cell counts and percentages as expected, there were no significant associations with these factors and plasma HHV8 levels. We identified increased proportions of CD8^+^ and CD4^+^ T cells expressing CD69 (P = 0.01 & P = 0.04 respectively), and increased CD57 expression on CD4^+^ T cells (P = 0.003) in participants with detectable HHV8.

**Conclusion:**

These results suggest there is a complex relationship between circulating HHV8 DNA and tissue-based disease in HIV-1 and HHV8 co-infected individuals with various malignancies.

## Introduction

Human Herpes Virus 8 (HHV8) is the causative agent of Kaposi’s Sarcoma (KS), a malignancy involving epithelial and other cells (*e*.*g*. B cells) of the lymphatic and circulatory systems. HHV8 infection alone is insufficient to cause clinical KS, and KS lesions are usually identified in individuals with significant immune dysregulation (*e*.*g*. HIV infection with low CD4^+^ T cell counts or immunosuppression following organ transplantation) [1,2]. KS can be an insidious disease with major associated morbidity, but may also improve clinically following recovery of CD4^+^ T cell counts or with specific chemotherapeutic intervention [3,4,5]. Iatrogenic KS is largely attributed to corticosteroid treatment, but B cell targeted therapies, such as rituximab, are also known to trigger HHV8 reactivation and the development of KS [6,7,8]. However, less is known about the significance of circulating levels of HHV8 without clinical KS in HIV infected individuals.

While it is well established that the tissue presence of HHV8 in combination with immunosuppression leads to the development of clinical KS, there are limited data on the dynamics of circulating HHV8 in HIV infected individuals receiving cytoreductive chemotherapies for various malignancies, or the impact of anti-tumor therapies on HHV8 viremia [3]. Furthermore, it is possible that circulating HHV8 may be associated with increased immune activation and exhaustion, but studies focusing on the associations between HHV8 viremia, CD4^+^ T cell counts, and lymphocyte phenotypes in the setting of HIV and malignancy are lacking. To address these issues, we examined the associations between different systemic cancer chemotherapies for KS and other malignancies with T cell counts and phenotype, circulating HHV8 DNA, HIV infection and clinical KS.

## Methods

Plasma and peripheral blood mononuclear cells (PBMCs) were obtained from a longitudinal cohort of HIV-infected individuals with a malignancy requiring systemic chemotherapy from 2012 until 2015 at Harvard Cancer-Center-affiliated institutions (Beth-Israel Deaconess Medical Center, Brigham and Women's Hospital/Dana-Farber Cancer Institute, and Massachusetts General Hospital). Whenever possible, whole blood samples were collected prior to initiation of chemotherapy, during chemotherapy and every six months thereafter. Longitudinal sampling was unavailable in some participants due to loss of follow-up, study adherence, or death. The Office for Human Research Studies of Dana-Farber/Harvard Cancer Center approved the study and written signed informed consent was obtained from each participant before sample collection.

Plasma was separated from cells and purified via double centrifugation, PBMC were isolated using density gradient centrifugation and cryopreserved until processing as previously described [9]. CD4^+^ T cells were depleted from total PBMC using the EasySep Human CD4^+^ T Cell Positive Selection kit (StemCell Technologies, Canada) prior to DNA extraction. Plasma and PBMC HHV8 DNA was extracted using Qiamp Blood Mini Kit (Qiagen, USA) following the manufacturer’s standard protocol.

Quantification of HHV8 DNA was performed on a LightCycler480 real-time PCR machine (Roche, USA) using a previously described method [10]. Results for plasma HHV8 DNA determination were adjusted by volume to obtain HHV8 DNA copies/mL; results of HHV8 DNA determination in CD4^+^ T cell-depleted PBMC HHV8 DNA results were normalized to cell counts based on quantitative PCR of the conserved human CCR5 gene as described [9,11]. IgG antibodies specific to HHV8 were measured by commercial immunofluorescence assay (Quest Diagnostics, Chantilly, VA) to provide an indication of prior HHV8 exposure.

Thawed, cryopreserved PBMCs were stained with Fixable Blue Dead Cell Stain, anti-CD3 (Invitrogen, USA), anti-CD4 (eBioscience, USA), anti-CD8, anti-CD38, anti-HLA-DR, anti-CD57, and anti-CD69 (BD Biosciences, USA). Following surface staining, cells were fixed, permeabilized and stained for intracellular anti-Ki-67 (BD). Samples were analyzed on a BD^TM^ LSRII flow cytometer using FACSDiva^TM^ software (BD) and FlowJo (Tree Star, Inc., USA). Statistical analyses were performed using Prism 5.0 (GraphPad Software). Non-parametric Mann Whitney U tests were used to identify significance between groups.

## Results

**Fig 1:**
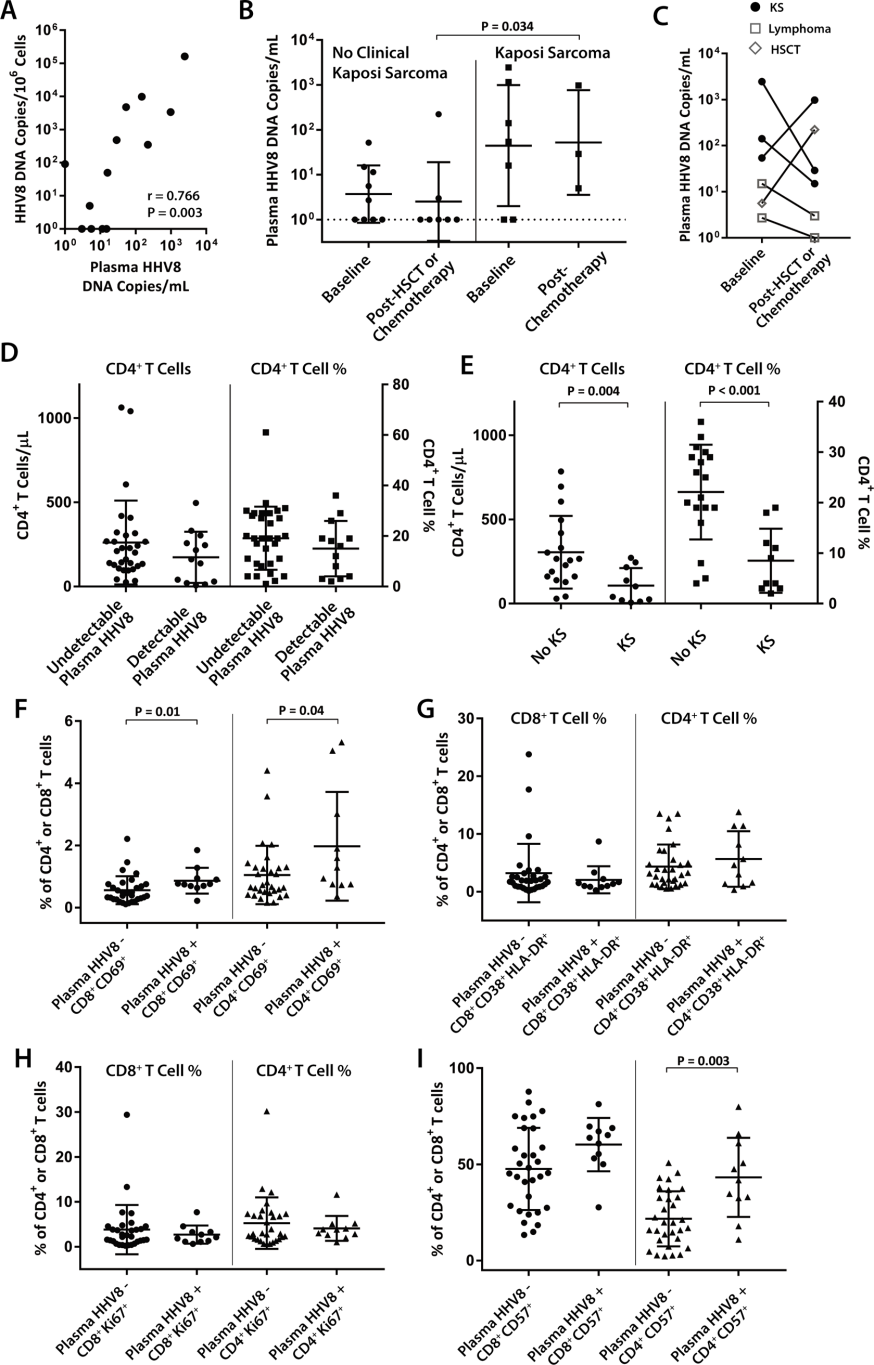
Relationships between circulating HHV8 DNA, Kaposi sarcoma (KS), and lymphocyte phenotypes in individuals with HIV-1 and concomitant malignancy. (**A**) Plasma and CD4^+^ T cell-depleted PBMC-associated HHV8 DNA are significantly and positively correlated by Spearman rank correlation analysis. (**B**) Individuals with clinical KS have higher plasma HHV8 DNA levels than those with other malignancies and evidence of prior HHV8 exposure (*e*.*g*. positive HHV8-specific antibodies or detectable HHV8 DNA), but there were no overall significant differences between pre- or post-chemotherapy time points by unpaired Mann Whitney U analysis. (**C**) Two individuals experienced >1-log_10_ increase in HHV8 DNA following treatment for KS or allogeneic stem cell transplantation for lymphoma. (**D**) No differences in CD4^+^ T cell counts or percentages were identified between participants with and without detectable circulating HHV8 DNA, despite significantly lower CD4^+^ T cell counts and percentages in individuals with clinical KS (**E**). Individuals with circulating HHV8 had significantly increased expression of CD69 (early activation marker) on both CD4^+^ and CD8^+^ T cells (**F**), but no differences in late markers of activation (**G**) or proliferation (**H**). (**I**) Significantly higher numbers of CD4^+^ T cells expressed CD57 (lymphocyte exhaustion marker) from individuals with circulating HHV8. P values calculated from Mann Whitney U analyses and data points represent all sample time points from all participants. HSCT = hematopoietic stem cell transplantation.

We analyzed samples from 29 HIV-1-infected individuals collected before initiation of cancer chemotherapy (or at the first collection time point during chemotherapy if no pre-treatment time point was available), and/or following completion of treatment. Of these individuals, 15 subjects were diagnosed with non-Hodgkin lymphoma (NHL), one diagnosed with acute myelogenous leukemia (AML), and one diagnosed with renal cell carcinoma (RCC). **Table 1**describes demographics, treatment history and HHV8 testing results for these participants with non-KS malignancies. Six participants were diagnosed with clinical KS (one had concomitant multicentric Castleman disease; MCD) as summarized in **Table 2**. Plasma and HHV8 levels at each collection time point for all study participants are shown in **S1 Table**. While 12 of 23 participants with non-KS malignancies (excluding HSCT recipients) received combination chemotherapy including rituximab, only one individual with KS/MCD received rituximab treatment alone. With the exception of six participants (two with active KS, and four with NHL), all patients were on combination antiretroviral therapy (ART) throughout this study and maintained HIV-1 viral loads of <1000 RNA copies/mL. One of the KS participants initiated ART post baseline sampling, and remained on ART throughout subsequent collection time points. 12 of 29 total participants, including all individuals with clinical KS had detectable anti-HHV8 antibodies. Six of these 12 individuals also had detectable HHV8 DNA. Interestingly, one participant with clinical KS had no detectable HHV8 DNA, and, of 15 individuals with NHL, four had detectable HHV8 DNA but no detectable anti-HHV8 antibodies. HHV8 DNA was detected in a higher number of plasma samples than in CD4^+^ T cell-depleted PBMC, although a significant positive correlation between plasma and cellular DNA copies was observed (P = 0.001; **Fig 1A**). No significant correlations were observed between plasma or cell-associated HHV8 DNA and plasma HIV-1 RNA.

**Table 1:** Patient Demographics, HHV8 DNA and Antibody Positivity, and clinical Details for participants with non-KS malignancies.

Pt	Sex	HHV8 Antibody	HHV8 DNA	ART at Time of Sampling	Clinical Diagnosis	Cancer Treatment
7	M	-	+	No	NHL	N/A
8^a^	F	-	-	No	NHL	R-CODOX-M/IVAC
9	M	-	+	Yes	NHL	R-EPOCH
10	M	-	+	Yes	NHL	R-CHOP
11	M	-	-	Yes	NHL	CHOP, GDP,
12	M	-	-	Yes	NHL	R-CHOP,
13	M	+	-	No	NHL	R-CHOP
14	M	-	-	Yes	NHL	R-CHOP
15^a^	M	+	+	Yes	NHL	R-EPOCH, DHAP
16	M	+	-	Yes	NHL	R-CHOP, R-ICE
17	M	+	-	Yes	NHL	R-EPOCH
18	M	-	-	No	NHL	EPOCH, R-ICE
19	M	-	-	-^b^	NHL	R-EPOCH
20	M	-	-	Yes	NHL	R-ICE
21	M	-	-	Yes	HL	ABVD
22	M	-	-	Yes	HL	ABVD
23	M	-	-	Yes	AML	Cytarabine-based
24	M	-	-	Yes	RCC	carboplatin/gemcitabine
25	M	-	-	Yes	HL	Autologous HSCT
26	M	+	-	Yes	HL	Allogeneic HSCT^c^
27	M	-	-	Yes	HL	Allogeneic HSCT^c^
28	M	+	-	Yes	HL	Allogeneic HSCT^c^
29^a^	Ma	-	+	Yes	NHL	Allogeneic HSCT^c^

Pt = participant; NHL = non-Hodgkin lymphoma; HL = Hodgkin’s Lymphoma; RCC = renal cell carcinoma; AML = acute myeloid leukaemia; R-EPOCH = rituximab, etoposiode, prednisone, vincristine, cyclophosphamide, doxorubicin; R-CODOX-M/IVAC = rituximab, cyclophosphamide, doxorubicin, vincristine, methotrexate/ifosfamide, etoposide, cytarabine; GDP = R-CHOP = rituximab, cyclophosphamide, doxorubicin, vincristine, prednisone; DHAP = dexamethasone, cytarabine, cisplatin; ABVD = doxorubicin, bleomycin, vinblastine, dacarbazine; HSCT = hematopoietic stem cell transplantation;

^a^ longitudinal data on detectable HHV8 levels were available

^b^ Unknown

^c^ Conditioning regimen included busulfan and fludarabine

**Table 2:** Patient Demographics, HHV8 DNA and Antibody Positivity, and clinical Details for participants with biopsy proven KS.

Pt	Sex	HHV8 Antibody	HHV8 DNA	ART at Time of Sampling	Clinical Details	Chemotherapy
1^a^	M	+	+	Yes^b^	Disseminated KS (skin, lung)	Paclitaxel/Doxorubicin
2	M	+	+	Yes	Extensive disseminated KS (skin, viscera, lung),	Paxlitaxel
3^a^	M	+	+	Yes	Disseminated cutaneous KS	Doxorubicin
4	M	+	-	Yes	Rectal mass and lymph node KS	Doxorubicin
5	M	+	+	Yes	Disseminated KS (skin, mucosa, pleura, lymph nodes)	Doxorubicin
6^a^	M	+	+	Yes	Cutaneous KS and concomitant MCD	Rituximab

Pt = participant; KS = Kaposi’s Sarcoma; MCD = multicentric Castleman disease

^a^ longitudinal data on detectable HHV8 levels were available

^b^ Patient started ART after baseline, pre-chemotherapy sampling

Overall, there were no significant differences between baseline and post-chemotherapy/HSCT plasma HHV8 DNA levels in individuals with or without baseline clinical KS. Of note, participants with clinical KS following cancer therapy had significantly higher levels of plasma HHV8 DNA than those with other malignancies (P = 0.034; **Fig 1B**). The higher levels of HHV8 DNA between pre-chemotherapy time points for participants with or without clinical KS were not statistically significant. Longitudinal sampling allowed paired analyses on six participants as shown in **Fig 1C**. Two individuals experienced increased HHV8 DNA following either KS-targeted chemotherapy or allogeneic HSCT for lymphoma. Interestingly, the increase in HHV8 DNA in the participant with clinical KS occurred in the setting of clinical improvement of disseminated lesions. In the HSCT recipient, HHV8 DNA was not detected in cells prior to HSCT, but rose to 9,888 copies/10^6^ CD4^+^ T cell-depleted PBMC 48 days following transplantation (**S1 Table**). In contrast, HIV DNA decreased from 12,363 copies/10^6^ CD4^+^ T cells at baseline to 6,695 copies/10^6^ CD4^+^ T cells following HSCT.

No significant differences in CD4^+^ T cell counts or percentages were identified between individuals with or without detectable plasma HHV8 DNA (**Fig 1D**). However, individuals with clinical KS had significantly lower mean CD4^+^ T cell counts and percentages than individuals without KS but with evidence of prior HHV8 exposure (*i*.*e*. HHV8-specific antibodies or detectable HHV8 DNA, **Fig 1E**).

Finally, significantly increased levels of CD69, an early activation marker, were identified on both CD8^+^ and CD4^+^ T cells in participants with detectable HHV8 compared to those without circulating DNA (P = 0.01 and P = 0.04, respectively; **Fig 1F**). However, no significant intergroup differences in the co-expression of CD38/HLA-DR (late activation markers) or Ki67 (intracellular proliferation marker) were observed (**Fig 1G and 1H**). CD57, a marker of immune exhaustion, was found to be significantly increased on CD4^+^, but not CD8^+^ T cells in participants with detectable HHV8 DNA (P = 0.003; **Fig 1I**).

## Discussion

Circulating HHV8 levels have been described as a predictor of clinical KS development [12,13], and all individuals receiving chemotherapy for KS or lymphoma in a prior study investigating HIV and malignancy reported decreased levels of circulating HHV8 in PBMC and plasma compared to baseline [3]. In our study, systemic cancer chemotherapy or transplantation had little consistent effect on circulating HHV8 burden. As a result, the relationship between circulating HHV8 DNA and clinical disease is likely more complex. For example, one participant in our study experienced improvement in disseminated KS lesions despite increasing circulating HHV8 DNA levels. Of note, a prior case report of an individual treated with rituximab, doxorubicin and valganciclovir, also demonstrated discordance between circulating HHV8 and clinical KS [14]. Furthermore, a HSCT recipient in our study with detectable HHV8 experienced a 4-log_10_ increase in HHV8 DNA copies/10^6^ cells 48 days following transplantation, despite an overall reduction in CD4^+^ T cell-associated HIV-1 DNA. It has been reported that allogeneic HSCT dramatically reduces HIV-1 DNA burden in the setting of ART following the development of full donor-cell chimerism [15], but the impact of HSCT on HHV8, including transplant conditioning and subsequent immune dysfunction, is underreported. While anecdotal, increasing HHV8 levels in our HSCT recipient may be a result of immune dysfunction following transplantation and persistent tissue sources of virus that escape donor cell replacement of the recipient hematopoietic compartment. Overall, four of five participants with either KS or lymphoma for whom longitudinal data was available experienced decreasing circulating HHV8 DNA levels. For example, the two participants with NHL and decreasing HHV8 DNA levels received doxorubicin as part of their chemotherapy for diffuse large B cell lymphoma, which has known activity against KS. Further study of HHV8 responses to various chemotherapeutic agents in larger cohorts of HIV-infected individuals is warranted. Rituximab, a monoclonal antibody that targets CD20 expressed on B cells, is thought to reactivate HHV8 viremia and lead to the development of clinical KS in both HIV-infected and uninfected individuals [16,17,18]. Interestingly, the participant with MCD/KS had a 1.9-log_10_ reduction in plasma HHV8 following rituximab monotherapy, but also recently started on suppressive antiretroviral therapy which may have led to improved anti-HHV8 immune responses.

In concordance with prior HIV-1 and lymphoma studies, we found that the prevalence of circulating HHV8 DNA in individuals with clinically evident KS was higher than in individuals with NHL (33%) [3]. In addition, four of ten individuals with detectable HHV8 DNA in our study did not have detectable anti-HHV8 antibodies as measured by commercial assay; discordance between HHV8 antibody testing and viral DNA has also been reported in HIV-1-infected individuals [19]. It is possible that antibody responses may be impaired due to immune suppression in the setting of concomitant HIV disease and malignancy. Despite detectable HHV8 in individuals with NHL, no individuals with Hodgkin’s lymphoma (HL) had detectable HHV8 DNA in plasma (two had detectable anti-HHV8 antibodies). Four of six of these individuals received stem cell transplant (three allogeneic and one autologous) for refractory disease and all completed systemic chemotherapy prior to sampling and HSCT. The lack of detectable HHV8 DNA in participants with HL may be a result of limited sample size or HSCT conditioning and donor-cell engraftment. As a result, longitudinal studies of HHV8 burden in a larger numbers of individuals with HL are needed.

The associations between plasma HHV8 DNA and markers of T cell activation, proliferation, and exhaustion in the setting of HIV and concomitant hematological malignancies have yet to be defined. We observed a greater frequency of CD4^+^ T cells expressing a marker of early immune activation from participants with circulating plasma HHV8, but no significant differences in expression of markers of late lymphocyte activation or T cell proliferation. However, significantly higher frequencies of CD4^+^ T cells expressing exhaustion markers were identified. These results are in the context of an HIV-1-infected population that typically has higher levels of immune activation and exhaustion that may have masked differences directly due to HHV8 or clinical KS.

In summary, this study demonstrates that concomitant cancer diagnosis in HIV infected individuals, chemotherapy and HSCT have variable associations with presence and levels of circulating HHV8, but that plasma HHV8 is associated with early markers of lymphocyte activation and exhaustion. Further study is therefore warranted to determine whether or not HHV8 viremia is a cause or effect of increased T cell activation and exhaustion, and to determine the long-term effects of circulating HHV8 DNA on clinical outcomes in HIV-infected individuals.

## Supporting information

S1 Table Plasma and HHV8 levels at each collection time point for all study participants. (DOCX)
