# Supplementary material for: Human Herpes Virus 8 in HIV-1 infected individuals receiving cancer chemotherapy and stem cell transplantation
Source: PLoS One. 2018 May 10;13(5):e0197298. doi: 10.1371/journal.pone.0197298 (PMC5944966; doi:10.1371/journal.pone.0197298)
Supplement: S1 Table — (DOCX) [file pone.0197298.s001.docx]

**S1 Table.** Plasma and HHV8 levels at each collection time point for all study participants

|  |  |  |  | Plasma HHV8 DNA (copies/mL) | | | | HHV8 (copies/10^6^ cells)^a^ | | |
| --- | --- | --- | --- | --- | --- | --- | --- | --- | --- | --- |
| HHV8 Ab+ | ART | Diagnosis | Chemotherapy | BL/OT | PT1 | PT2 | PT3 | BL/OT | PT1 | PT2 |
| 0 | No | NHL | N/A | 52 |  |  |  |  |  |  |
| 1 | Yes | KS | Paclitaxel/Doxorubicin | 141 | ND | <15 |  | ND | 91 | 5 |
| 0 | No | NHL | MIVAC |  | ND | ND |  |  | ND | ND |
| 0 | Yes | NHL | R-EPOCH | 11.4 |  |  |  | ND |  |  |
| 0 | Yes | NHL | R-CHOP | 2.7 | ND | ND |  | ND | ND | ND |
| 1 | Yes | KS/MCD | Rituximab | 2453 | 29 |  |  | 161339 | 483 |  |
| 0 | Yes | NHL | CHOP, GDP, MTX |  | ND |  |  |  | ND |  |
| 0 | Yes | HL | ABVD | ND | ND | ND | ND | ND | ND | ND |
| 0 | Yes | NHL | R-CHOP, MTX |  | ND | ND | ND | ND | ND |  |
| 1 | No | NHL | R-CHOP | ND | ND |  |  | ND | ND |  |
| 0 | Yes | NHL | R-CHOP | ND | ND | ND |  | ND | ND | ND |
| 1 | Yes | KS | paxlitaxel | 16 |  |  |  | 50 |  |  |
| 1 | Yes | NHL | EPOCH, DHAP | 15 | 3 |  |  | ND |  |  |
| 1 | Yes | KS | Doxorubicin | 54 | 976 |  |  | 4798 | 3363 |  |
| 1 | Yes | NHL | R-CHOP, R-ICE | ND |  |  |  | ND |  |  |
| 1 | Yes | NHL | R-EPOCH | ND | ND | ND | ND | ND | ND |  |
| 0 | No | NHL | EPOCH, R-ICE | ND | ND |  |  | ND | ND |  |
| 1 | Yes | KS | Doxorubicin | ND |  |  |  | ND |  |  |
| 0 | Yes | HL | ABVD | ND | ND |  |  | ND |  |  |
| 0 | Yes | RCC | Carboplatin/Gemcitabine | ND | ND |  |  | ND | ND |  |
| 0 | Unknown | NHL | R-EPOCH | ND |  |  |  |  |  |  |
| 1 | Yes | KS | Doxorubicin | 1163 |  |  |  |  |  |  |
| 0 | Yes | NHL | R-ICE | ND |  |  |  |  |  |  |
| 0 | Yes | HL | Autologous HSCT | ND | ND |  |  | ND | ND | ND |
| 1 | Yes | HL | Allogeneic HSCT |  | ND | ND |  |  |  |  |
| 0 | Yes | HL | Allogeneic HSCT |  | ND | ND |  |  |  |  |
| 1 | Yes | HL | Allogeneic HSCT |  | ND | ND |  |  |  |  |
| 0 | Yes | NHL | Allogeneic HSCT | 5.6 | 150 | 222 |  | ND | 9888 | 351 |
| 0 | Yes | AML | R-EPOCH/CHOP |  | ND |  |  |  |  |  |
|  | | | | | | | | | | |

KS = Kaposi’s Sarcoma; MCD = multicentric Castleman disease; NHL = non-Hodgkin lymphoma; HL = Hodgkin’s Lymphoma; RCC = renal cell carcinoma; AML = acute myeloid leukaemia; R-EPOCH = rituximab, etoposiode, prednisone, vincristine, cyclophosphamide, doxorubicin; R-CODOX-M/IVAC = rituximab, cyclophosphamide, doxorubicin, vincristine, methotrexate/ifosfamide, etoposide, cytarabine; GDP = R-CHOP = rituximab, cyclophosphamide, doxorubicin, vincristine, prednisone; DHAP = dexamethasone, cytarabine, cisplatin; ABVD = doxorubicin, bleomycin, vinblastine, dacarbazine; HSCT = hematopoietic stem cell transplantation; BL/OT = baseline or first collection time point at the start of chemotherapy; PT = post-treatment (chemo/HSCT) timepoints

^a^ Cells were not available for some sample time point during which plasma was collected for HHV8 testing
